# Predictive markers of obesity and glucose metabolism dysfunction in adult common marmosets (*Callithrix jacchus*)

**DOI:** 10.1038/s41366-025-01841-2

**Published:** 2025-07-25

**Authors:** Juan Pablo Arroyo, Corinna N. Ross, Jessica Greig, Ricki J. Colman, Suzette D. Tardif, Michael L. Power

**Affiliations:** 1 Southwest National Primate Research Center, Texas Biomedical Institute, San Antonio, TX USA; 2 Wisconsin National Primate Research Center, Madison, WI USA; 3 Department of Cell & Regenerative Biology, School of Medicine and Public Health, University of Wisconsin, Madison, WI USA; 4 Center for Species Survival, Smithsonian’s National Zoo and Conservation Biology Institute, Washington, DC USA

**Keywords:** Metabolism, Physiology

## Abstract

**Objective:**

Characterize the effects of obesity on common marmoset glucose metabolism and develop predictive markers of glucose metabolism dysfunction.

**Methods:**

Body size, weight, lean mass, fat mass, %fat, resting energy expenditure (REE), and glycosylated hemoglobin (HbA1c) were measured on 51 adult marmosets. Physical activity was assessed using actimeter collars (*n* = 50). A body mass-per-length parameter (BML) was constructed. Animals were classified as without obesity or with obesity (%fat >10%) and by the age they obtained maximum weight (Maxwt). Correlation, MANOVA, and binary logistic regression were used to examine relationships between parameters; path analysis to explore directional relationships.

**Results:**

Body fat and BML were correlated (*r* = 0.565, *p* < 0.001). Both were correlated with HbA1c (*r* = 0.658; *r* = 0.764, *p* < 0.001). Activity was negatively correlated with %fat and REE (*r* = −0.437, *p* = 0.002; *r* = −0.473, *p* < 0.001). REE was correlated with %fat, BML, and HbA1c (*r* > 0.5, *p* < 0.001). Marmosets with obesity were more likely to have elevated HbA1c (>5.7%; odds ratio = 8.25, *p* = 0.003). BML above 3.4 g/mm predicted obesity (OR = 6.25 [95% CI 1.62–24.02], *p* = 0.008) and high HbA1c (OR = 29.47 [95% CI 6.21–139.72], *p* < 0.001). Early Maxwt predicted increased fat mass (*F* = −0.476, *p* = 0.015) and high %fat (*F* = −0.084, *p* = 0.014).

**Conclusion:**

Both %fat and BML were markers for high HbA1c. Early maximum adult weight predicts increased adiposity and risk of glucose dysfunction.

## Introduction

Common marmosets (*Callithrix jacchus*) are small South American nonhuman primates (NHP) that are an important biomedical and basic science model in a variety of biomedical research areas including neuroscience, aging, and infectious disease studies [1–3]. Marmosets offer many advantages over other NHP models. They are small, typically weighing between 300–450 grams, they mature rapidly, becoming reproductively competent at around 1.5 years of age, are considered aged at 8–12 years old, and can produce litters of 2–3 offspring every 5–6 months [4, 5]. Demand for common marmosets has increased at an extraordinary rate over the past 10 years due to their emergence as a critical model organism [1, 6]. Like other NHP, their genetic similarity to humans provides great translational value [7].

Obesity accompanied by dyslipidemia and compromised glucose metabolism is emerging as a frequent clinical finding in marmosets housed in captivity [8–10]. Mean body mass in marmoset colonies has been steadily increasing and many colonies now report mean body mass above 400 g [3]. Previous research has suggested that in adult marmosets, body fat above 10% is an appropriate threshold for obesity [11]. Marmosets in the colony at the Southwest National Primate Research Center (SNPRC) have a high prevalence of obesity, particularly in sub-adult marmosets ranging between 1–2 years of age with 46–52% of animals in this age category exhibiting obesity and reduced insulin sensitivity [12]. Our previous research has shown that marmosets at risk of obesity can be detected within the first 6 months of life [11] and that early obesity is associated with glucose metabolism dysfunction [13].

Obesity has generated increases in pathologies rarely seen previously in marmosets, such as hepatomegaly, hepatic steatosis, diabetes, atherosclerosis, cardiomyopathies, and stroke [9]. While these conditions generate considerable interest in marmosets as a model of obesity and metabolic syndrome, its value is limited by our incomplete understanding of what has led to the increases in spontaneous obesity observed in so many marmoset colonies. It is still not clear whether differences in care between institutions may produce unintended variation in body size, weight, body composition, physical activity, or blood glucose levels. A deeper understanding of the relationships between these variables, could potentially turn marmoset obesity from a poorly understood and spontaneous phenomenon, into a condition tightly controlled by researchers.

It also remains unclear how metabolic parameters such as resting energy expenditure (REE) (i.e., amount of energy used to maintain essential physiological functions at rest), relate to variability in physical activity and phenotypic variation such as body composition. Marmosets are thought to require diets high in metabolizable energy; however, the increasing recognition of obesity in marmoset colonies [9, 10] may indicate an overestimation of energy requirements. Kleiber’s [14] estimated daily mammalian metabolic rate is 70 × (body mass (kg))^0.75^, and the National Research Council (NRC) recommends 2 × the estimated metabolic rate as the maintenance energy requirement for captive nonhuman primates (140 × (body mass(kg))^0.75^) [15]. However, our previous study of 81 marmosets at two institutions fed three different diets found that digestible energy intake (DEI) had a mean value of approximately 1.5 times the Kleiber estimate for metabolic rate although it varied widely among individuals of the same body mass [16]. Studies of resting metabolic rate (RMR) in related species (e.g., golden lion tamarins, pygmy marmosets, and Goeldi’s monkeys) suggest that marmoset RMR likely is below the Kleiber estimate [17–21]. These studies suggest that marmoset energy requirements are substantially lower than the recommended amount.

In this study we assessed body weight, morphometrics, body composition, physical activity, fasting blood glucose, glycated hemoglobin (HbA1c), and resting energy expenditure (REE) in captive marmosets between 2 and 7 years of age housed at the Southwest National Primate Research Center. The objectives of this study were to investigate relationships between body composition, morphometric measurements of body size, REE, activity, and glucose metabolism and to develop markers predicting glucose metabolism dysfunction in marmosets.

## Methods

This study examined 51 early-mid adult (2–7 years of age) common marmosets (*Callithrix jacchus*), of which 26 were males and 25 were females. The sample is composed by animals originating from the Southwest National Primate Research Center (SNPRC) (*n* = 35) and from the New England Primate Research Center (NEPRC) (*n* = 16). At the time of data collection (2018–2019), all subjects were housed at the SNPRC, Texas Biomedical Research Institute, an AAALAC accredited institution. Marmosets from the NEPRC were imported to SNPRC between 2014 and 2015 and had been integrated into the SNPRC colony for 3 to 5 years. At SNPRC, all marmosets in this study were maintained according to the standardized husbandry conditions as previously described [22]. Animals receive two base diets, the Harlan Teklad marmoset purified diet, and the Mazuri marmoset diet [23]. Marmosets are provided with daily food enrichment consisting of small quantities of various rotating items, including dried cranberries, fresh fruit, and legumes. Water is available ad libitum through a water bottle with ball tip sipper tube system. Food intake is not restricted; however overall consumption is observed daily, and body weight is monitored on a regular basis.

During this project, animals were housed as single, male-female pairs, or family groups consisting of the breeding male and female and their offspring. Females were regularly cycling and analyses were not limited to any particular part of the reproductive cycle. None of the females were pregnant, and none of subjects had been involved in previous studies that would affect the measured parameters of this study. The research protocols were approved by the SNPRC animal care and use committee (IACUC #1519 CJ), adhered to the American Society of Primatologists (ASP) Principles for the Ethical Treatment of Non-Human Primates, and complied with all applicable U.S. laws regarding animal research. All the research meets the journal’s ethical guidelines, including adherence to the legal requirements of the study country.

Animals enrolled on the study were weighed by placing a scale in the cage and rewarding them for getting onto the scale. Longitudinal data on body mass was extracted from electronic medical records to determine the age at which maximum body mass was achieved outside of pregnancy. Body size (morphometrics) was assessed with digital calipers (linear measurements, e.g. suprasternal-pubic length) and measuring tape (circumference measurements) using a standardized set of length and circumference measures used previously [24], with all measures made in triplicate, and then averaged. Measures of fat and lean mass were performed via quantitative magnetic resonance (QMR) with EchoMRI (Houston, Texas, USA) [25] as previously described [11]. Briefly, unsedated animals were placed in the EchoMRI QMR for a 2-min scan. For blood glucose measurements, food was retrieved at 4 p.m. and animals were fasted overnight for 16–17 h prior to testing at 8–9 a.m. A fasted blood sample was collected via femoral venipuncture after placing the marmoset in the Wisconsin restraint device and analyzed for HbA1c using Siemens Healthineers DCA Vantage Analyzer: HemoglobinA1c cartridge (Erlangen, Germany) and glucose using Bayer Contour Next glucometer (Leverkusen, Germany). A Camntech Actiwatch mini actimeter (Fenstanton, United Kingdom) was placed on a fitted collar for 1 week of data collection.

Resting energy expenditure (REE) was measured twice for all animals (*n* = 51) during daytime (between 9 a.m. and 3 p.m.) over a 2-week period with a Sable Systems FoxBox (Las Vegas, Nevada, USA) flow-through respirometry system. Animals were fasted for two hours and placed in a nestbox within a metabolic chamber with controlled air flow rate (1000 ml/min) and temperature (28.9 °C) for 2 h of data collection. Oxygen consumption (VO_2_ mlO_2_/h) was calculated as VO_2_ = 60 * Flow Rate * [(Baseline O_2_ – Mean O_2_) / 100] / (1 − 0.2095). Carbon dioxide production (VCO_2_ mlCO_2_/h) was calculated as VCO_2_ = 60 * Flow Rate * (Mean CO_2_ – Baseline CO_2_) / 100. A modified Weir equation [26] was used to calculate REE in kcal/day (REE = 5.46 * VO_2_ + 1.75 * VCO_2_) / 60).

Data were analyzed with R version 4.1 [27] in an open-source statistical spreadsheet jamovi version 2.3.17 [28] and with IBM SPSS (Armonk, New York, USA). Multivariate analysis of variance (MANOVA) was employed to examine differences by institution of origin, by sex, between animals with obesity and without obesity, and between early and late achievers of maximum weight. Associations between measured parameters were assessed by correlation analysis.

Marmosets were labeled “without obesity” if percent body fat was less than 10% and “with obesity” if body fat was 10% or greater [11]. In a previous study of 20 marmosets (10 male and 10 female) all animals above 400 g body mass exceeded 10% body fat [24]. Accordingly, we also analyzed the data using the categories body mass of 400 g and less and above 400 g. A mass-by-length parameter (BML) was constructed by dividing body mass by suprasternal-pubic length (SSPL) with units of g/mm and its associations with percent body fat and HbA1C were examined by correlation. A categorical parameter for BML was constructed based on piecewise linear regression of HbA1c against BML with a breakpoint determined at 3.4 g/mm. Differences in mean values of other parameters between these categories were assessed by ANOVA and presented in Table 1.

**Table 1:** Effects of high body fat, high weight, and high body mass-per-length on other measured parameters (*n* = 51).

Measured parameter	Less than 10% body fat	10% body fat or greater	*F* (*η*²*p*)	*p* value	400 g and below	Above 400 g	*F* (*η*²*p*)	*p* value	Less than 3.4 g/mm	3.4 g/mm and above	*F* (*η*²*p*)	*p* value
% Body fat	5.2 ± 0.6 (3.4)	13.7 ± 1.0 (3.7)	NA	NA	3.8 ± 0.8 (3.5)	10.4 ± 0.8 (4.4)	32.7 (0.400)	<0.001	5.2 ± 0.8 (4.4)	11.0 ± 0.9 (4.5)	21.3 (0.303)	<0.001
Weight (g)	401.1 ± 13.9 (83.2)	517.0 ± 18.1 (70.2)	22.4 (0.314)	<0.001	343.7 ± 8.1 (37.0)	499.2 ± 12.1 (66.2)	NA	NA	369.7 ± 10.3 (55.3)	521.6 ± 13.1 (61.4)	85.8 (0.637)	<0.001
Fasting glucose (mg/dL)	110.8 ± 9.0 (54.2)	127.4 ± 9.6 (38.1)	1.16 (0.023)	0.286	105.3 ± 10.6 (48.5)	122.9 ± 9.3 (50.9)	1.54 (0.031)	0.220	108.3 ± 10.7 (57.7)	125.4 ± 8.0 (37.3)	1.46 (0.029)	0.233
HbA1c (%)	5.5 ± 0.3 (1.6)	6.8 ± 0.4 (1.4)	7.74 (0.136)	0.008	4.9 ± 0.3 (1.3)	6.5 ± 0.3 (1.6)	14.4 (0.227)	<0.001	5.1 ± 0.3 (1.6)	6.8 ± 0.3 (1.3)	15.9 (0.245)	<0.001
Activity (movement/30 s)	81.3 ± 7.6 (44.8)	60.6 ± 7.0 (27.2)	2.76 (0.054)	0.103	92.5 ± 10.7 (47.7)	63.5 ± 5.9 (32.0)	6.63 (0.121)	0.013	82.3 ± 8.5 (45.2)	65.9 ± 7.3 (34.2)	2.0 (0.040)	0.164
Resting energy expenditure (kcal/day)	30.6 ± 1.1 (6.7)	40.1 ± 2.3 (9.0)	17.3 (0.261)	<0.001	27.3 ± 1.1 (5.0)	37.6 ± 1.5 (8.0)	27.0 (0.355)	<0.001	28.7 ± 1.0 (5.1)	39.6 ± 1.8 (8.3)	34.0 (0.410)	<0.001
Body mass-per-length (g/mm)	3.03 ± 0.08 (0.5)	3.57 ± 0.11 (0.4)	13.5 (0.216)	<0.001	2.68 ± 0.07 (0.33)	3.54 ± 0.06 (0.35)	76.9 (0.611)	<0.001	2.8 ± 0.06 (0.3)	3.7 ± 0.05 (0.24)	NA	NA
Suprasternal-Pubic (mm)	132.2 ± 1.8 (11.0)	144.6 ± 1.8 (6.8)	16.2 (0.248)	<0.001	128.4 ± 1.5 (6.8)	141.1 ± 2.0 (11.2)	21.3 (0.303)	<0.001	132.1 ± 1.7 (9.1)	140.7 ± 2.7 (12.5)	8.14 (0.143)	0.006
Supersternal-Pubic (mm)	91.3 ± 1.8 (11.0)	101.4 ± 2.4 (9.3)	9.78 (0.166)	0.003	88 ± 2.0 (9.0)	98.7 ± 2.0 (11.0)	13.8 (0.219)	<0.001	91.4 ± 1.9 (10.0)	98 ± 2.6 (12.3)	4.46 (0.084)	0.040
Knee-Heel (mm)	71.4 ± 0.63 (3.8)	73.6 ± 0.8 (3.0)	4.03 (0.076)	0.05	71.1 ± 0.9 (4.2)	72.8 ± 0.6 (3.1)	2.73 (0.053)	0.105	71.6 ± 0.7 (3.7)	72.7 ± 0.8 (3.6)	1.18 (0.024)	0.283
Chest Circumference (mm)	152 ± 2.4 (14.1)	172.9 ± 4.13 (16.0)	21.5 (0.305)	<0.001	143.1 ± 1.5 (7.0)	168.7 ± 2.7 (14.5)	55.7 (0.532)	<0.001	147.7 ± 2.2 (11.8)	172 ± 2.9 (13.6)	46.9 (0.489)	<0.001
Abdominal Circumference (mm)	129.9 ± 3.6 (21.7)	159.6 ± 4.9 (18.8)	21.4 (0.304)	<0.001	116.7 ± 2.96 (13.6)	154 ± 3.4 (18.5)	61.7 (0.557)	<0.001	122.9 ± 3.0 (16.3)	159.5 ± 3.8 (17.7)	58.6 (0.545)	<0.001
Thigh Circumference (mm)	88.1 ± 1.37 (8.2)	93.6 ± 1.7 (6.5)	5.25 (0.097)	0.026	82.8 ± 1.3 (6.0)	94.6 ± 1.0 (5.5)	52.8 (0.519)	<0.001	84.7 ± 1.2 (6.5)	96.3 ± 1.0 (4.5)	51.5 (0.512)	<0.001

Values are mean ± SEM (SD).

Based on human recommendations, values for HbA1c were considered “normal” if less than 5.7% and “high” if 5.7% or higher. The association between percent body fat and weight categories and HbA1c category was assessed using Chi-square. The associations between the mass-to-length (BML) index with body fat and HbA1c categories were assessed by Chi-square.

Binary logistic regression was used to assess whether body mass and BML can be used as indices for obesity and glucose metabolism dysfunction in marmosets. Specifically, we examined whether body mass above 400 g and/or BML greater than 3.4 g/mm were useful predictors of obesity (defined as body fat above 10%) or glucose metabolism dysfunction (HbA1c above 5.7%).

### Path analysis

After relationships between variables were identified with regression analysis we employed path analysis, an extension of regression, that allows testing different models of how variables are connected to each other. As such, path analysis allows testing for directional effects of individual connections (paths) between the variables included in a model [29]. Path analysis models were conducted with R packages lavaan [30] and semPlot [31] in jamovi module PATHj [32]. Path models were produced to investigate how weight, age at maximum weight, body size, body composition, and physical activity relate to each other and contribute to resting energy expenditure. Model fit for path analysis was assessed with Chi-square goodness of fit test, root mean square error of approximation (RMSEA), comparative fit index (CFI), Tucker-Lewis index (TLI) and standardized root mean square residual (SRMR) [33].

## Results

There were no significant differences between sexes in any measured parameter. Accordingly, data from males and females were combined in the analysis (Supplementary Table 1). There were no significant differences in weight, circumference morphometrics, lean body mass (LBM), REE, fasting glucose or HbA1c for animals with different institutional origins. However, animals born at SNPRC in this study were younger (*M* = 3.48, SD = 1.1) than those born at NEPRC (*M* = 5.37, SD = 0.94; *F* = 33.64, *p* < 0.001), achieved maximum weight at an earlier age (SNPRC: *M* = 49.9, SD = 16.1; NEPRC: *M* = 78.7, SD = 17; *F* = 33.0, *p* < 0.001), had longer bodies (i.e., higher suprasternal-pubic (SNPRC: *M* = 138, SD = 11.53; NEPRC: *M* = 130, SD = 9.25); and knee-heel lengths (SNPRC: *M* = 72.8, SD = 3.84; NEPRC: *M* = 70.4, SD = 2.63; *F* = 6.09, *p* = 0.02)), had higher adiposity (i.e., higher fat mass (SNPRC: *M* = 43.7, SD = 30.4; NEPRC: *M* = 22.6, SD = 22.7; *F* = 7.04, *p* = 0.01), fat percentage (SNPRC: *M* = 8.96, SD = 5.06; NEPRC: *M* = 4.96, SD = 4.53; *F* = 8.49, *p* = 0.005), and fat-to-lean mass ratio (SNPRC: *M* = 0.11, SD = 0.07; NEPRC: *M* = 0.06, SD = 0.06; *F* = 7.31, *p* = 0.01)), and were less active (SNPRC: *M* = 61, SD = 36.5; NEPRC: *M* = 105, SD = 34.7; *F* = 15.87, *p* < 0.001) than animals born at NEPRC.

Two animals with extremely high fasting glucose (269 mg/dL and 314 mg/dL) and the highest HbA1c values (above 10%) are now deceased. On necropsy one was classified with diabetes; full necropsy data are not available for the other animal. Analyses done with and excluding these marmosets returned the same qualitative results for most parameters. Accordingly, we excluded these two animals from analyses except where noted. Their results are included in the mean values in Table 1 and identified in all Figures.

Calculated daytime resting energy expenditure (REE) was correlated with body mass (*r* = 0.681, *p* < 0.001). Compared to the Kleiber estimate, REE in marmosets averaged 88.8 ± 2.2% of the expected value. After accounting for body mass, the only other parameter related to REE was a negative correlation with activity (*r* = −0.387, *p* < 0.001). Activity was negatively correlated with most measures of body size (e.g. body mass, SSPL, percent body fat). Smaller, leaner marmosets were generally more active than larger marmosets (Table 1).

**Fig. 1:**
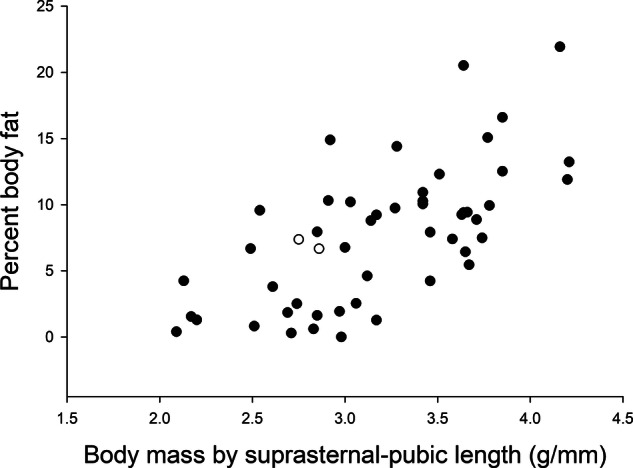
The relationship between body fat% and body mass divided by suprasternal-pubic length (BML). The two marmosets with diabetes indicated by open circles. BML explained 43.1% of the adjusted variance in body fat%. For every one-unit increase in BML (g/mm), the value of body fat% was predicted to increase by 6.38 (95% CI 4.32–8.44) *p* < 0.001.

**Fig. 2:**
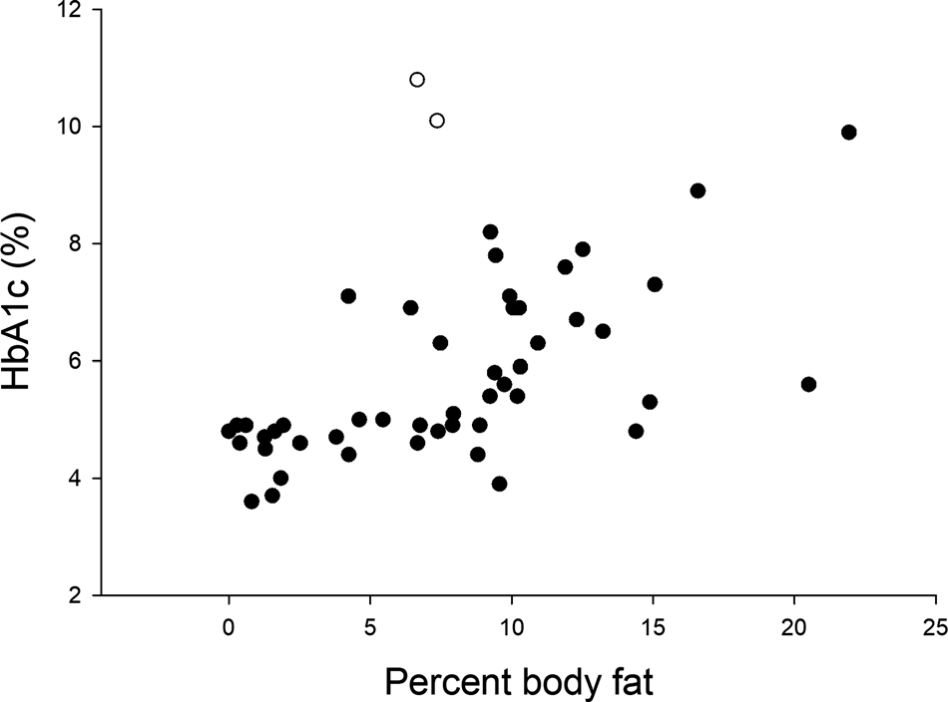
The relationship between body fat% and glycosylated hemoglobin (HbA1c). The two marmosets with diabetes indicated by open circles. Body fat% explained 26.3% of the adjusted variance in HbA1c. For every one-unit increase in body fat%, the value of HbA1c was predicted to increase by 0.169 (95% CI 0.091–0.248) *p* < 0.001.

**Fig. 3:**
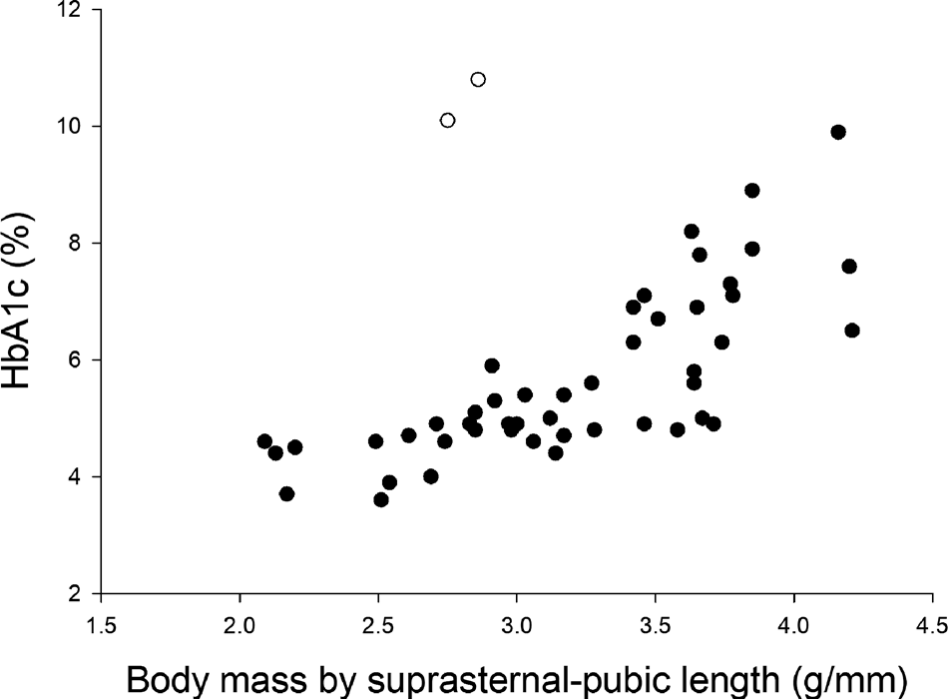
The relationship between HbA1c and body mass divided by suprasternal-pubic length (BML). The two marmosets with diabetes indicated by open circles. BML explained 28.3% of the adjusted variance in HbA1c. For every one-unit increase in BML (g/mm), the value of HbA1c was predicted to increase by 1.678 (95% CI 0.937–2.42) *p* < 0.001.

There were strong associations between percent body fat and BML (*r* = 0.565, *p* < 0.001, including marmosets with diabetes, Fig. 1), and between HbA1c and fasting glucose (*r* = 0.751, *p* < 0.001, including marmosets with diabetes, Supplementary Fig. 1). Percent body fat and BML were correlated with HbA1c (animals with diabetes not included; *r* = 0.658, *p* < 0.001, Fig. 2, and *r* = 0.764, *p* < 0.001, Fig. 3, respectively). The relationships between fasting glucose and BML and percent body fat were not significant if animals with diabetes were included but were significant when these animals were excluded (*r* = 0.464, *p* < 0.001 and *r* = 0.347, *p* = 0.015, respectively). Age at max weight, weight, fat mass, lean mass, and abdominal circumference cumulatively explained 43% of the variance in HbA1c. After controlling for age at max weight, weight, fat mass, and lean mass, we found that higher abdominal circumference predicted higher HbA1c, *F*(5,45) = 8.57, Adj. *R*^2^ = 0.431, *p* < 0.001; with every one-unit (mm) increment in abdominal circumference predicting an increase in the value of HbA1c by 0.04679 (*p* = 0.018).

Among all 51 marmosets, 30 (58.8%) were above 400 g body mass, 15 (29.4%) had obesity, 20 (39.2%) had high (above 5.7%) HbA1c values, and 22 (43.1%) had a BML value above 3.4 g/mm. There were 11 (21.6%) exceptionally lean marmosets (body fat below 2%, as measured by QMR). Compared with the other 25 marmosets without obesity (below 10% body fat) these marmosets had lower body mass and LBM, but not smaller SSPL. They also had lower HbA1C values (4.5 ± 0.1% versus 5.4 ± 0.3%). Otherwise, they did not differ from marmosets with between 2 and 10% body fat in fasting glucose, age, activity, or REE (after accounting for body mass).

### Above 400 g body mass

Marmosets above 400 g body mass had higher percent body fat, BML, and HbA1c, and lower measures of activity (all *p* < 0.001; Table 1). A greater proportion of high-weight marmosets had HbA1c values above 5.7% (58.6% versus 5.0%; *p* < 0.001). Marmosets above 400 g body mass were more likely to have body fat above 10% (48.3% versus 5.0%) and BML above 3.4 g/mm (75.9% versus 0.0%, all *p* < 0.001). Examining the 30 marmosets above 400 g, only 8 (26.7%) had both HbA1c values below 5.7% and percent body fat below 10%. That number drops to 4 (13.3%) if BML under 3.4 g/mm is also included. In contrast, for marmosets under 400 g body mass (excluding animals with diabetes) all 20 had a BML under 3.4 g/mm and 19 of 20 had HbA1c below 5.7% and percent body fat less than 10%. The single animal under 400 g with percent body fat over 10% had an HbA1c value of 5.9%.

### Above 10% body fat

Marmosets with obesity were larger (higher weight, SSPL), had higher HbA1c and BML values, and lower activity (all *p* < 0.001; Table 1). A greater proportion of marmosets above 10% body fat had HbA1c values above 5.7% (73.3% versus 25.0%, respectively, *p* = 0.001). Logistic regression also indicated they were more likely to have elevated HbA1c (*B* = 2.11, OR = 8.25, [95% CI 2.095–32.488], *p* = 0.003).

### BML greater than 3.4 g/mm

Marmosets with BML 3.4 g/mm and above were larger, had higher adiposity, and higher HbA1c (all *p* < 0.001; Table 1). A greater proportion of marmosets with a BML above 3.4 g/mm had greater than 10% body fat (50.0% versus 13.8%, respectively, *p* = 0.005) and HbA1c values above 5.7% (77.3% versus 10.3%%, respectively, *p* < 0.001).

Among the 29 marmosets without diabetes above 400 g, 22 (75.9%) had a BML above 3.4 g/mm. The seven animals above 400 g with BML below 3.4 g/mm all had HbA1c values below 5.7%. Of the 22 marmosets above both 400 g and 3.4 g/mm 17 (77.3%) had HbA1c values above 5.7%.

### Binary logistic regression

The results of the binary logistic regressions support both body mass above 400 g and BML greater than 3.4 g/mm as indices of obesity and glucose metabolism dysfunction in marmosets. Mass above 400 g predicted obesity with 66.7% accuracy (*B* = 2.86, OR = 17.5 [95% CI 2.07–147.62], *p* = 0.009) and glucose dysfunction with 72.5% accuracy (*B* = 2.66, OR = 14.25 [95% CI 2.79–72.70], *p* = 0.001). A value of BML above 3.4 g/mm predicted obesity with 70.6% accuracy (*B* = 1.83, OR = 6.25 [95% CI 1.62–24.02], *p* = 0.008) and glucose dysfunction with 84.3% accuracy (*B* = 3.38, OR = 29.47 [95% CI 6.21–139.72], *p* < 0.001).

### Obtaining maximum weight

**Fig. 4:**
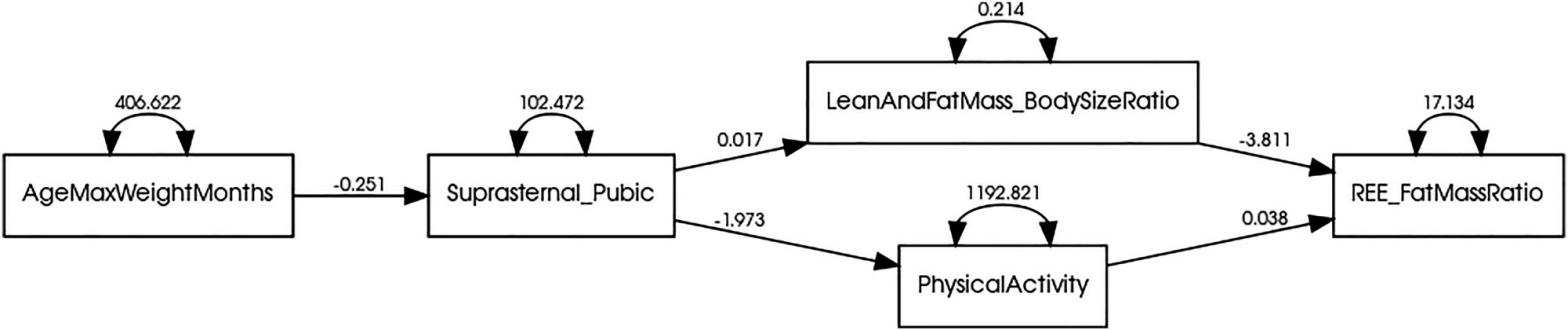
Node diagram for the path analysis model. Marmosets that achieved maximum body mass earlier, ended up with a larger body size (*B* = −0.25, *p* < 0.001). Larger body size predicted higher body mass in proportion to body size (*B* = 0.02, *p* = 0.003), and lower activity (*B* = −1.97, *p* < 0.001). In turn, higher body mass in proportion to body size (*B* = −3.81, *p* = 0.002) and lower activity (*B* = 0.04, *p* = 0.01), predicted lower REE per gram of fat mass. Additional statistics available in Table 2.

Early achievers of maximum body mass (i.e., before the average age of 58.9 months) reached a larger final body size (*F* = 9.35, *p* = 0.004), were less active (*F* = 9.61, *p* = 0.003), had higher fasting glucose (*F* = 5.34, *p* = 0.025) and HbA1c values (*F* = 7.72, *p* = 0.008), and also exhibited higher fat mass, however, differences in fat mass did not reach statistical significance (*F* = 4.02, *p* = 0.051). Age at maximum body mass explained 9.85% of the variance in body fat percentage, with the earlier achievement of maximum body mass predicting increased body fat percentage (*B* = −0.084, *F* = 6.46, *p* = 0.014). Large (above mean) body size increased the odds of obesity, with 13 out of the 15 animals with obesity also exhibiting large bodies (*B* = 2.56, OR = 13.0 [95% CI 2.52–67.16], *p* = 0.002). In the path model, animals that achieved maximum body mass earlier, ended up with a larger body size (*B* = −0.25, *p* < 0.001). Larger body size predicted higher body mass in proportion to body size (*B* = 0.02, *p* = 0.003), and lower activity (*B* = −1.97, *p* < 0.001). In turn, higher body mass in proportion to body size (*B* = −3.81, *p* = 0.002) and lower activity (*B* = 0.04, *p* = 0.01), predicted lower REE per gram of fat mass (Table 2 and Fig. 4).

**Table 2:** Path analysis model with age at maximum body mass, final body size, body mass to body size ratio, physical activity, and resting energy expenditure proportional to fat mass.

Parameter Estimate	Unstandardized	Standardized	*p*
Regressions			
AgeMaxWeightMonths → Suprasternal_Pubic	−0.25 (0.07)	−0.45	<0.001
Suprasternal_Pubic → LeanAndFatMass_BodySizeRatio	0.02 (0.006)	0.39	0.003
Suprasternal_Pubic → PhysicalActivity	−1.97 (0.44)	−0.54	<0.001
LeanAndFatMass_BodySizeRatio → REE_FatMassRatio	−3.81 (1.20)	−0.39	0.002
PhysicalActivity → REE_FatMassRatio	0.04 (0.01)	0.31	0.010
Errors			
Error in AgeMaxWeightMonths	406.62 (82.15)	1.00	<0.001
Error in Suprasternal_Pubic	102.47 (20.70)	0.80	<0.001
Error in LeanAndFatMass_BodySizeRatio	0.21 (0.04)	0.85	<0.001
Error in PhysicalActivity	1192.82 (240.99)	0.71	<0.001
Error in REE_FatMassRatio	17.13 (3.46)	0.70	<0.001

*χ*^2^(5) = 6.67, *p* = 0.246. Unstandardized loadings (standard errors), standardized loadings, and significance levels for each parameter in the path analysis model.

## Discussion

Assessments for detecting obesity in non-human primates typically rely on indirect measures of adiposity. These include proportional measures of total body mass to body size (i.e., lengths and circumferences) [34], body mass ratios between captive and wild populations [35], and subjective semi-quantitative valuations such as body condition scoring [36]. Previously, we identified 10% body fat as an appropriate threshold for obesity in marmosets [11]. Here we tested relationships between BML (body mass-to-length), direct measures of adiposity via QMR, and glucose dysfunction. As result, we identified a marmoset-specific BML threshold for obesity.

Our study results confirm earlier published findings that adult marmosets with body fat above 10% are at risk of obesity-related diseases, specifically problems with glucose regulation [11]. In addition, BML the body mass-per-length measurement using the suprasternal-pubic length was a good indicator of high body fat with normal marmosets having values below 3.4 g/mm. A BML over 3.4 g/mm was an even better indicator of glucose metabolism dysfunction, defined as HbA1c values above 5.7%. The biological and clinical significance of the 3.4 g/mm BML threshold is based on its ability to identify individuals with obesity with 70.6% accuracy and those with glucose dysfunction with 84.3% accuracy. Body mass above 400 g in marmosets may also be a marker of overweight and potential glucose metabolism issues. Very few marmosets under 400 g body mass had high body fat or high HbA1c values, indicating that 400 g is generally a healthier body mass for marmosets. However, there were some marmosets above 400 g with healthy profiles as defined by percent body fat, HbA1c value and BML, suggesting that there are some “large” healthy marmosets.

Body mass and suprasternal-pubic length (SSPL) are reliable parameters that marmoset colony managers should be able to track consistently to provide a biomarker of risk of obesity and metabolic health. Indeed, once a marmoset has achieved full growth SSPL should be relatively constant, with variation being more likely due to measurement error. Thus, once a marmoset has become adult the first SSPL measurement taken can be used and then only body mass needs to be tracked in order to calculate BML. We suggest that a BML value above 3.4 g/mm would serve as an easy index to identify common marmosets at risk for obesity-related glucose metabolism dysfunction. Although this study was limited to marmoset data, comparison with human metrics may offer useful context for interpreting obesity classifications across species. In marmosets, a BML threshold greater than 3.4 g/mm identified individuals with obesity (defined as >10% body fat) with a sensitivity of 73.3%. By contrast, a meta-analysis of 31,968 individuals across 32 studies found that using a BMI ≥ 30 kg/m² to define obesity in humans yielded a sensitivity of only 42%, indicating that a substantial proportion of individuals with excess body fat were not correctly identified [37].

Potential confounders among the assessed variables included institution of origin, age and sex. Although there were differences between institutions (i.e., age, age at maximum weight, final body size, adiposity, and physical activity), institution of origin did not predict obesity status, weight above 400 g, BML above 3.4 g/mm, or HbA1c above 5.7%. Similarly, sex and age did not predict obesity status, weight above 400 g, BML above 3.4 g/mm, or HbA1c above 5.7%.

We found no differences between the sexes. Common marmosets are generally considered sexually monomorphic, a trait that may be linked to the evolution of small body size and cooperative breeding [38]. Female marmosets previously have been reported to exhibit significantly higher body weight, fat mass, and fat-lean mass ratio than males in captivity [10]. However, the absence of statistically significant differences in body size, weight, energy expenditure (REE), and body composition between females and males in the present study support the notion that captive common marmosets either lack [13, 24, 39] or exhibit minimal sexual dimorphism [3, 9, 40], and that these small differences were not impacted by the institution where the marmosets were housed. Although marmosets born at SNPRC were younger than marmosets born at NEPRC, all animals had reached final adult size and were not aged (i.e., aging-related decline ≥8 years) at the time of assessment. Therefore, age differences by institution did not explain any measured parameter.

In humans, rapid postnatal growth is associated with increased risk for obesity, high blood pressure, and impaired glucose tolerance [41–45]. A meta-analysis encompassing 18,576 participants from 20 studies, reported that higher fat percentage during childhood and adolescence is associated with higher fasting plasma glucose, fasting plasma insulin, and insulin resistance [46]. Marmosets exhibit a similar trend with early weight gain and glucose metabolism. In marmosets, accelerated gain in body mass during early-life results in an increased risk of excess adiposity during the first year of life [11]. The results of the present study strongly suggest that early attainment of maximum body mass in marmosets may lead to a larger body size, reduced physical activity, higher adiposity, and impaired glucose metabolism (i.e., higher fasting glucose and HbA1c values).

Marmosets that developed obesity at an early age also displayed accelerated developmental traits, such as becoming independent earlier and beginning to eat solid food at an earlier age [12]. They also displayed different eating behavioral phenotypes, ingesting larger quantities of liquid food per each lick and a tolerance for foods with a higher fat content that marmosets without obesity would avoid [12]. Further research in the marmoset model is warranted to investigate relationships between early-life accelerated mass gain and hormones (e.g., ghrelin, leptin, insulin, cortisol, glucagon-like peptide-1, adiponectin, etc.) involved in appetite regulation, feeding behavior, and the development of obesity-related metabolic dysregulations.

It remains unclear whether differences related to early achievement of maximum body mass and large body size were produced via developmental plasticity or due to variance in early-life food intake. Maternal effects have also been implicated. Maternal exposure to a high-fat diet, and maternal body fat have been independently associated with the production of offspring with higher birth weights [11]. Moreover, offspring postnatal exposure to a high-fat diet results in increased body fat and lean mass, but with higher adiposity (i.e., higher fat-lean mass ratio) at 6 months [11]. Increased dietary intake of carbohydrate or fat, in captive adult marmosets, has also been shown to result in altered cardiometabolic function and changes in body composition [9]. Alternatively, founder’s effect at the establishment of this captive population, followed by unintentional selection of animals that undergo rapid growth and achieve larger bodies might explain these differences. In this sense, rapid growth and early-life survival might have been favored due to increased reproductive success, possibly at the expense of healthspan and lifespan [47].

While it is well known that many of the characteristics that we evaluated are often correlated, we were interested in evaluating how body composition was associated with glucose metabolism. After controlling for other factors, we found that higher abdominal circumference predicted higher HbA1c. This finding suggests that visceral fat and abdominal subcutaneous fat likely contribute to higher HbA1c values. In humans, visceral fat mass has been reported to have a stronger association with insulin resistance, than total fat mass or abdominal circumference [48]. However, this could not be tested in the present study as we performed whole body composition analysis via quantitative magnetic resonance (QMR), and did not quantify visceral fat or subcutaneous fat at the abdomen. To corroborate whether visceral fat contributes to higher HbA1c in marmosets, future studies should assess visceral fat, as well as subcutaneous fat deposition at distinct segments of the body with imaging techniques (e.g., DXA, MRI, CT).

In this observational study, we identified predictive markers of obesity and glucose metabolism dysfunction in marmosets and found that many of the measured parameters were strongly interrelated. Using path analysis, we incorporated multiple correlated variables to construct a conceptual model representing plausible relationships among these factors, informed by the chronological sequence of events and their clinical relevance [29]. While path analysis does not establish causality, its diagrammatic output can be used to generate testable hypotheses for future experimental research. Statistical control helped to isolate the effects of individual variables and account for potential confounders. Nonetheless, controlled experimental studies are necessary to determine whether causal relationships exist among early attainment of maximum body mass, large body size, reduced physical activity, obesity, and glucose metabolism dysfunction in marmosets.

## Supplementary information

Supplemental Figure 1 Legend

Supplemental Figure 1

Supplemental Table 1

## Data Availability

The data that support the findings of this study are available from the authors upon reasonable request.
