# Supplementary material for: Predictive markers of obesity and glucose metabolism dysfunction in adult common marmosets (Callithrix jacchus)
Source: Int J Obes (Lond). 2025 Jul 25;49(10):2011–8. doi: 10.1038/s41366-025-01841-2 (PMC12532565; doi:10.1038/s41366-025-01841-2)
Supplement: Supplementary file 1 — Supplemental Figure 1 Legend [file 41366_2025_1841_MOESM1_ESM.docx]

Supplemental Figure 1. The relationship between fasting glucose and glycosylated hemoglobin (HbA1c). The two marmosets with diabetes indicated by arrows. Fasting glucose explained 55.6% of the adjusted variance in HbA1c. For every one-unit increase in fasting glucose (mg/dL), the value of HbA1c was predicted to increase by 0.025 (95% CI 0.019-0.031) p < 0.001.
