# Supplementary material for: Predictive markers of obesity and glucose metabolism dysfunction in adult common marmosets (Callithrix jacchus)
Source: Int J Obes (Lond). 2025 Jul 25;49(10):2011–8. doi: 10.1038/s41366-025-01841-2 (PMC12532565; doi:10.1038/s41366-025-01841-2)
Supplement: Supplementary file 3 — Supplemental Table 1 [file 41366_2025_1841_MOESM3_ESM.docx]

Supplemental Table 1. Data for all subjects. Glucose and HbA1c data for the two diabetic marmosets are in bold.

| ID | Sex | Age  (months) | Mass (g) | Fat  (g) | SSPL  (mm) | VO_2_  (mlO_2_/hr) | VCO_2_  (mlCO_2_/hr) | Glucose  (mg/dL) | HbA1C  (%) |
| --- | --- | --- | --- | --- | --- | --- | --- | --- | --- |
| Cj1 | F | 77.2 | 552 | 51.9 | 151.7 | 368.8 | 312.1 | 95 | 5.8 |
| Cj2 | F | 75.2 | 539 | 34.7 | 147.6 | 290.2 | 244.6 | 191 | 6.9 |
| Cj3 | M | 56.8 | 409 | 36.0 | 130.2 | 258.5 | 224.0 | 77 | 4.4 |
| Cj4 | F | 48.9 | 545 | 51.4 | 148.8 | 366.1 | 314.8 | 146 | 7.8 |
| Cj5 | F | 48.8 | 380 | 28.0 | 138 | 270.3 | 224.9 | **269** | **10.1** |
| Cj6 | M | 43.2 | 540 | 53.6 | 142.8 | 324.6 | 286.2 | 123 | 7.1 |
| Cj7 | M | 80.3 | 358 | 0.0 | 120.2 | 219.2 | 213.3 | 140 | 4.8 |
| Cj8 | F | 76.6 | 458 | 50.0 | 133.9 | 363.6 | 373.7 | 165 | 6.3 |
| Cj9 | M | 75.5 | 357 | 1.0 | 131.8 | 186.8 | 185.5 | 81 | 4.9 |
| Cj10 | M | 57.9 | 370 | 7.1 | 124.4 | 211.5 | 170.8 | 77 | 4.9 |
| Cj11 | M | 61.2 | 483 | 48.5 | 141.2 | 367.5 | 304.6 | 143 | 6.9 |
| Cj12 | M | 60.5 | 361 | 6.7 | 134.2 | 280.2 | 206.4 | 91 | 4 |
| Cj13 | F | 59.0 | 406 | 10.3 | 132.8 | 250.0 | 200.1 | 92 | 4.6 |
| Cj14 | M | 48.2 | 409 | 36.3 | 110.2 | 259.6 | 238.3 | 105 | 4.9 |
| Cj15 | F | 47.2 | 346 | 2.1 | 122.1 | 251.3 | 230.0 | 113 | 4.9 |
| Cj16 | M | 50.4 | 520 | 78.4 | 138 | 342.1 | 288.8 | 127 | 7.3 |
| Cj17 | F | 51.0 | 529 | 54.3 | 154.5 | 311.5 | 283.6 | 116 | 6.9 |
| Cj18 | M | 36.7 | 386 | 4.9 | 121.9 | 205.7 | 194.2 | 90 | 4.7 |
| Cj19 | M | 71.5 | 415 | 17.6 | 119.8 | 274.2 | 246.0 | 86 | 7.1 |
| Cj20 | M | 79.4 | 486 | 35.9 | 135.8 | 297.1 | 254.6 | 75 | 4.8 |
| Cj21 | M | 76.7 | 289 | 4.5 | 132.9 | 269.2 | 209.6 | 93 | 3.7 |
| Cj22 | F | 68.9 | 461 | 21.3 | 147.9 | 241.6 | 198.2 | 74 | 5 |
| Cj23 | F | 63.6 | 453 | 35.8 | 130.8 | 372.8 | 262.4 | 105 | 4.9 |
| Cj24 | F | 54.5 | 366 | 5.9 | 128.4 | 314.7 | 253.5 | 72 | 4.8 |
| Cj25 | M | 47.6 | 304 | 2.5 | 121.1 | 192.5 | 160.1 | 82 | 3.6 |
| Cj26 | F | 33.7 | 387 | 35.7 | 121.9 | 187.4 | 176.9 | 72 | 5.4 |
| Cj27 | F | 32.9 | 322 | 8.1 | 117.4 | 204.9 | 197.6 | 97 | 4.6 |
| Cj28 | F | 29.8 | 387 | 26.2 | 128.96 | 191.3 | 187.5 | 205 | 4.9 |
| Cj29 | M | 41.2 | 557 | 92.4 | 144.6 | 416.8 | 364.1 | 198 | 8.9 |
| Cj30 | M | 57.2 | 405 | 27.0 | 141.6 | 273.4 | 231.3 | **311** | **10.8** |
| Cj31 | M | 54.2 | 546 | 50.5 | 150.6 | 293.8 | 272.2 | 101 | 8.2 |
| Cj32 | F | 49.7 | 618 | 73.5 | 147 | 282.8 | 246.9 | 178 | 7.6 |
| Cj33 | F | 50.1 | 421 | 62.7 | 144.2 | 310.2 | 266.1 | 85 | 5.3 |
| Cj34 | M | 50.1 | 451 | 46.0 | 148.6 | 285.3 | 257.5 | 83 | 5.4 |
| Cj35 | F | 56.3 | 508 | 104.2 | 139.7 | 268.9 | 234.3 | 83 | 5.6 |
| Cj36 | M | 45.3 | 466 | 45.4 | 142.3 | 276.6 | 273.3 | 128 | 5.6 |
| Cj37 | M | 25.7 | 420 | 22.9 | 114.3 | 189.3 | 168.8 | 106 | 5 |
| Cj38 | M | 37.7 | 590 | 73.9 | 153.4 | 408.6 | 328.8 | 160 | 7.9 |
| Cj39 | F | 39.2 | 484 | 69.7 | 147.6 | 270.5 | 232.9 | 80 | 4.8 |
| Cj40 | F | 36.1 | 613 | 134.5 | 147.3 | 433.5 | 366.1 | 159 | 9.9 |
| Cj41 | F | 35.3 | 593 | 78.5 | 140.7 | 406.4 | 359.2 | 122 | 6.5 |
| Cj42 | M | 35.0 | 308 | 11.7 | 117.8 | 230.2 | 218.5 | 72 | 4.7 |
| Cj43 | F | 34.8 | 375 | 29.8 | 131.6 | 226.4 | 188.2 | 122 | 5.1 |
| Cj44 | M | 34.8 | 388 | 40.0 | 133.4 | 248.3 | 246.3 | 108 | 5.9 |
| Cj45 | F | 34.3 | 341 | 22.8 | 136.7 | 270.1 | 244.1 | 72 | 4.6 |
| Cj46 | M | 30.9 | 558 | 41.8 | 149.2 | 341.9 | 322.7 | 70 | 6.3 |
| Cj47 | F | 27.6 | 295 | 3.8 | 134.3 | 259.3 | 228.0 | 90 | 4.5 |
| Cj48 | M | 26.1 | 324 | 31.0 | 127.5 | 193.2 | 163.5 | 71 | 3.9 |
| Cj49 | F | 25.6 | 285 | 12.1 | 133.63 | 353.5 | 259.6 | 108 | 4.4 |
| Cj50 | F | 25.3 | 542 | 66.7 | 154.5 | 306.0 | 245.8 | 104 | 6.7 |
| Cj51 | M | 25.3 | 288 | 1.13 | 138.1 | 267.4 | 218.9 | 86 | 4.6 |
| mean |  | 48.9 | 435.2 | 37.1 | 135.8 | 284.0 | 246.7 | 115.7 | 5.8 |
| SEM |  | 2.3 | 13.3 | 4.2 | 1.6 | 9.1 | 7.6 | 7.0 | 0.2 |
